# Severe Delayed-Onset Meningitis Developed One Year After a Basilar Skull Fracture Without a Cerebrospinal Fluid Leak: A Case Report

**DOI:** 10.7759/cureus.75261

**Published:** 2024-12-07

**Authors:** Aya Ozaki, Takamitsu Iwata, Eisaku Terada, Ryuichiro Kajikawa, Takashi Tsuzuki, Haruhiko Kishima

**Affiliations:** 1 Department of Neurosurgery, Sakai City Medical Center, Sakai, JPN; 2 Department of Neurosurgery, Osaka University Graduate School of Medicine, Suita, JPN

**Keywords:** basilar skull fracture, cerebrospinal fluid leak, delayed-onset meningitis, head trauma, ventriculitis

## Abstract

Traumatic cerebrospinal fluid (CSF) leakage from skull base fractures increases the risk of bacterial meningitis, which is associated with a high mortality rate in adults, and commonly results in severe neurological outcomes. While most cases of CSF leakage occur within three months post-injury and generally resolve spontaneously, delayed-onset meningitis remains a challenging complication. Herein, we report a rare case of severe bacterial meningitis with an intraventricular abscess one year following a frontal skull base fracture, despite no CSF leak.

A 68-year-old man with a history of a frontal skull base fracture due to a motor vehicle accident presented with seizures, fever, and altered consciousness. Imaging revealed ventriculitis, while laboratory findings confirmed bacterial meningitis caused by *Streptococcus pneumoniae*. Following initial antibiotic therapy, imaging revealed a new abscess near the fracture site, indicating potential bacterial entry due to a dural injury. Surgical repair was performed using a periosteal pedicle flap to close the intracranial space from the paranasal sinus. Postoperatively, antibiotic treatment resolved the abscesses, and the patient was subsequently treated for postmeningitis hydrocephalus. This case underscores the risk of delayed-onset meningitis in patients with large skull base fractures, even those without CSF leaks. In cases of skull base trauma with bone defects, a potential occult dural injury should be considered. If meningitis develops, surgical dural repair should be evaluated in addition to antibiotic treatment, regardless of the presence of a CSF leak, to effectively manage the infection and prevent a poor prognosis.

## Introduction

Traumatic cerebrospinal fluid (CSF) leakage occurs following the tearing of the dura mater due to head trauma, leading to rhinorrhea or otorrhea [1]. Between 7% and 30% of cases of traumatic CSF leaks can lead to bacterial meningitis [2-7]. The mortality rate of bacterial meningitis in adults is approximately 20%, while patients commonly suffer from significant neurological sequelae, resulting in an overall poor prognosis [8]. Traumatic CSF leakages occur in 10-30% of skull base fractures [9,10], and they typically present within 48 hours of injury, with most cases occurring within three months [11]. Traumatic CSF leakage often resolves spontaneously in the early post-injury period [12]. Consequently, only cases with persistent CSF leakage have been managed through surgical dural repair, such as craniotomy or transnasal approaches [1]. However, there are reports of delayed-onset meningitis in cases where CSF leakage initially resolved spontaneously [12]. Due to the unpredictable occurrence of delayed post-traumatic meningitis, as well as the potential for a severe clinical course, it poses a significant clinical challenge.

Herein, we present a rare case of severe bacterial meningitis with an intraventricular abscess that developed one year following a frontal skull base fracture, despite the absence of a CSF leakage. The patient underwent dural repair using a pedicled periosteal flap, resulting in a favorable outcome.

## Case presentation

**Figure 1:**
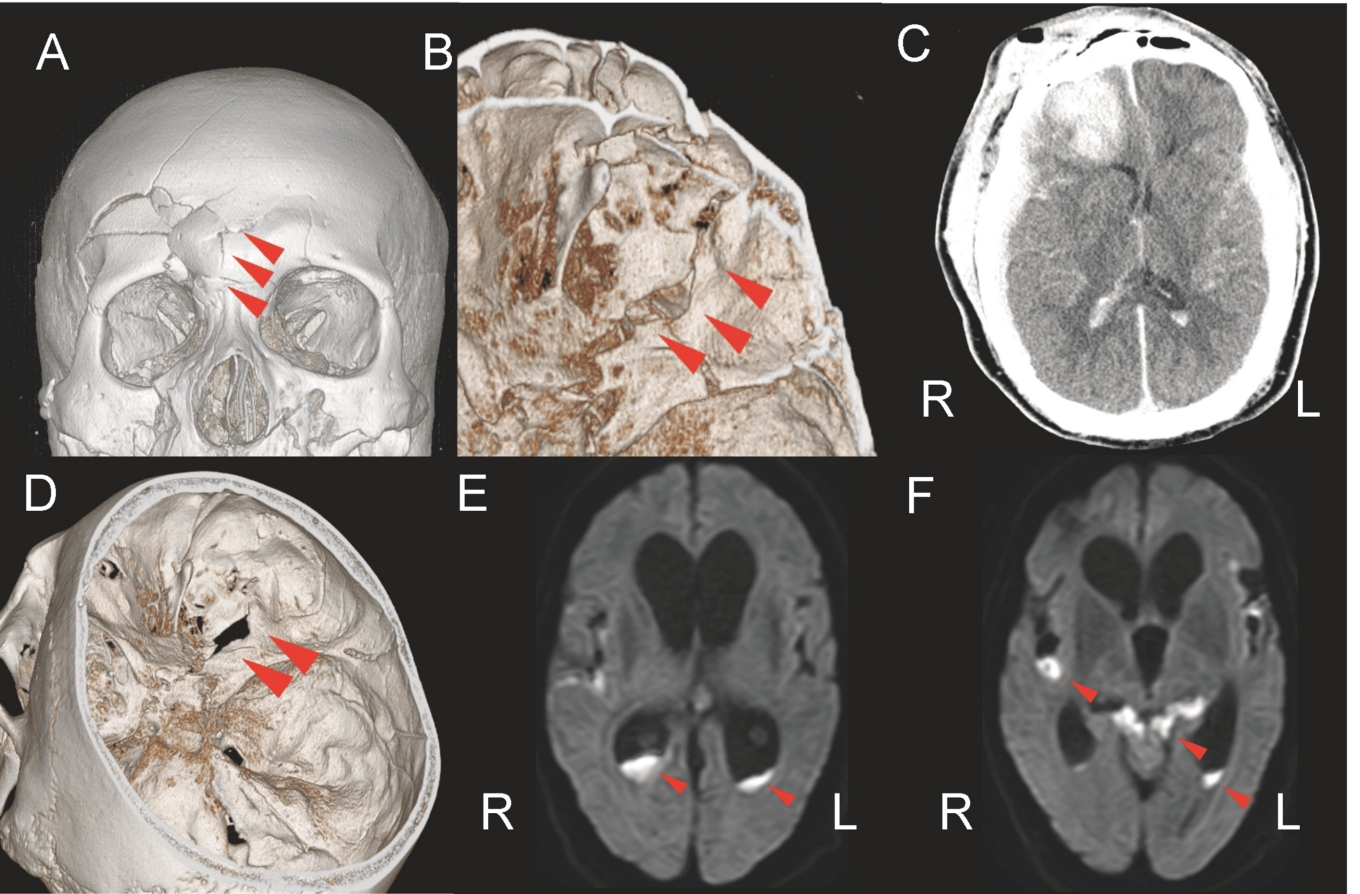
Imaging findings in head trauma and delayed onset meningitis. A: Frontal view of the initial three-dimensional CT image at the time of head trauma. Red arrows indicate the fracture site. B: Superior view of the right frontal skull based on 3D CT scan images at the time of head trauma. The red arrows indicate the fracture line and bony defect. C: Axial CT tomography following head trauma showing an intraparenchymal hematoma within the right frontal lobe contusion. D: 3D CT image at the start of meningitis treatment, showing the view from above the skull base. E: Axial DWI image at meningitis onset. Red arrows indicate the high-intensity areas in both of the lateral ventricles. F: Axial DWI image at the onset of meningitis. Red arrows indicate high-intensity areas within the ventricles and subarachnoid spaces. DWI: diffusion-weighted imaging

The patient was a 68-year-old man with a medical history of a motor vehicle accident one year prior. The patient’s past medical history included diabetes mellitus and hypertension. The accident resulted in severe traumatic injuries. The patient had a contusion on the right forehead, and blood was present in the nasal cavity, but no external injuries were observed on the body trunk. The patient’s Glasgow Coma Scale (GCS) score was E3V4M6, with no abnormalities in the pupillary light reflex or signs of paralysis. A head CT scan revealed a right frontal lobe contusion and frontal skull base fracture (Figures 1A-1C), with no evidence of subdural pneumocephalus. At that time, the patient did not exhibit CSF leakage. Due to hemodynamic instability, priority was given to stabilizing his vital signs through abdominal hemostasis for intra-abdominal hemorrhage under general anesthesia with tracheal intubation. A follow-up head CT scan revealed an increase in the hematoma size in the right frontal lobe contusion with a midline shift. An intracranial pressure (ICP) sensor was placed, and the ICP remained below 10 mmHg without additional surgery. A tracheotomy tube was placed seven days after admission. Three months postoperatively, the patient received conservative treatment and was transferred to a long-term care facility with a modified Rankin Scale (mRS) score of 4. A head CT scan before discharge showed a small amount of air in the epidural space, but there was no fluid retention in the paranasal sinuses.

One year after the accident, the patient developed seizures and was hospitalized at a different institution, at which antiseizure medication was initiated. Following hospitalization, he experienced a fever and altered consciousness. Magnetic resonance imaging (MRI) revealed hyperintense areas indicative of ventriculitis on diffusion-weighted imaging (DWI), leading to a suspicion of severe bacterial meningitis. The patient was subsequently referred to our department for further evaluation and treatment.

On admission, the patient’s GCS score was E1V2M4. His vital signs showed a blood pressure of 129/92 mmHg, a heart rate of 94 bpm, and a body temperature of 36.6°C. Laboratory results further revealed a white blood cell count of 131,800/µL and a C-reactive protein level of 35.6 mg/dL. Examination of the CSF revealed a yellow, turbid fluid with a total nucleated cell count of 193,141/µL and a glucose level of 2 mg/dL. CT scan images of the head revealed bone separation at the frontal skull base with ventricular enlargement (Figure 1D). MRI revealed hyperintense areas in the lateral ventricles and subarachnoid space on DWI, indicative of abscesses, as well as hyperintense areas in the mastoid air cells and paranasal sinus on fluid-attenuated inversion recovery (FLAIR) imaging (Figures 1E, 1F).

Clinical course

**Figure 2:**
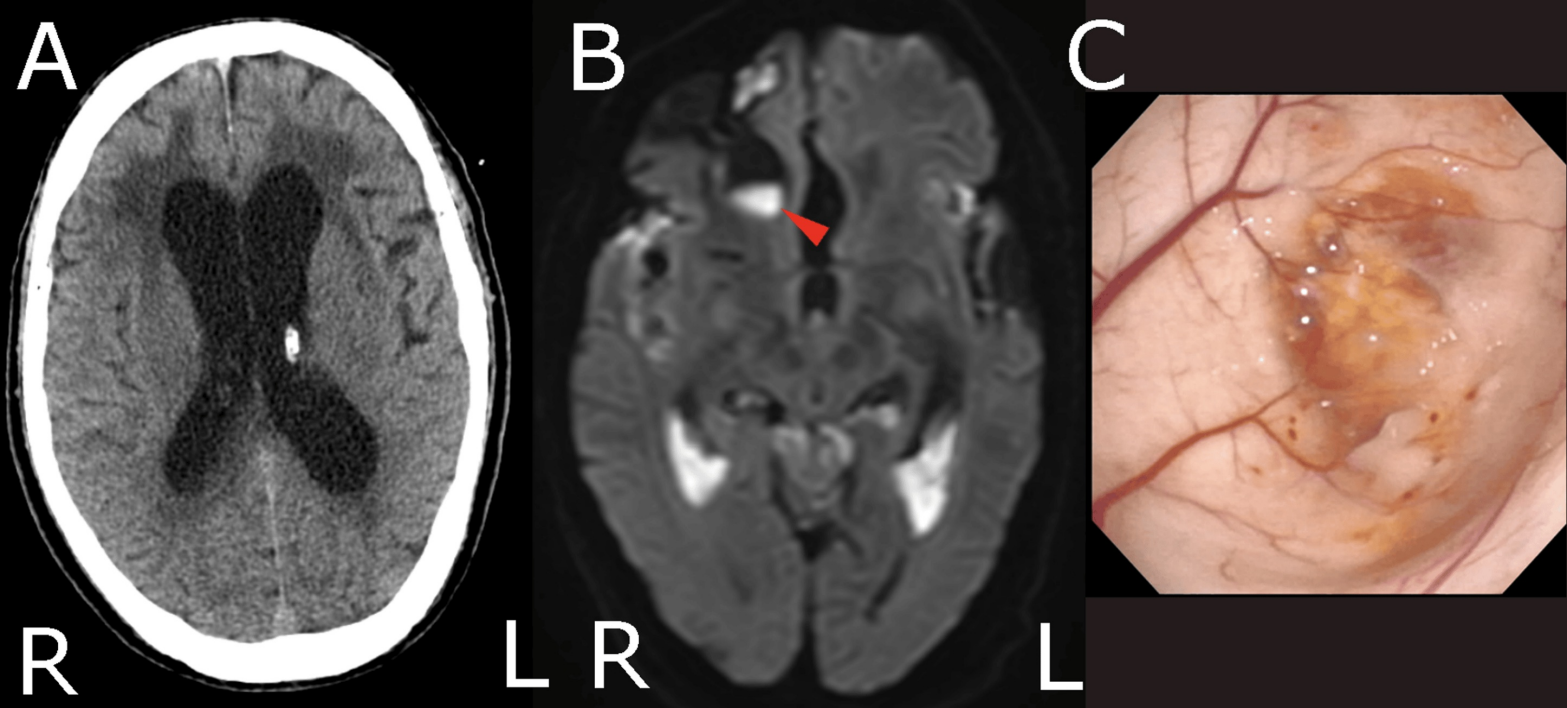
Imaging findings after admission and intraoperative endoscopic observations. A: Axial CT scan following ventricular drainage. B: Axial DWI image obtained on the fifth day of hospitalization. The red arrow indicates a new high-signal area in the right anterior horn of the lateral ventricle. C: Endoscopic observation of the right anterior horn of the lateral ventricle. The ependymal wall was partially defective, and an abscess invading the ventricle was observed through the ependymal vessels. DWI: diffusion-weighted imaging

Based on these findings, the patient was diagnosed with severe bacterial meningitis and ventriculitis. On the first day of treatment, 6 grams per day of meropenem and 1.8 grams per day of vancomycin were infused. Further, a ventricular drain was placed, as shown in Figure 2A. On the third day, *Streptococcus pneumoniae *was identified using the FilmArray meningitis/encephalitis panel (BioFire Diagnostics, LLC, Salt Lake City, USA), prompting a switch from meropenem to 4 grams per day of ceftriaxone. Vancomycin dosing was adjusted according to the trough levels. While inflammatory markers in the blood gradually decreased, a new hyperintense area, indicative of an abscess, appeared in the anterior horn of the right lateral ventricle on DWI performed on day 5 (Figure 2B). Given that the newly detected abscess was located above the site of the frontal skull base fracture and frontal lobe contusion, we suspected that damage to the dura led to communication between the paranasal sinuses and subdural space.

When we replaced the ventricular drain to avoid drain infection on day 8, an endoscopic examination of the right lateral ventricle was performed. Observation of the anterior horn of the right lateral ventricle revealed a partial defect in the ependymal wall, and pus was visible beyond the vascular structures of the ependyma (Figure 2C). This finding led us to conclude that bacteria had entered the lateral ventricle via the infected contused brain and that antibiotics alone would be insufficient for definitive treatment. Therefore, we decided to perform dural repair to close communication between the paranasal sinuses and intracranial space.

**Figure 3:**
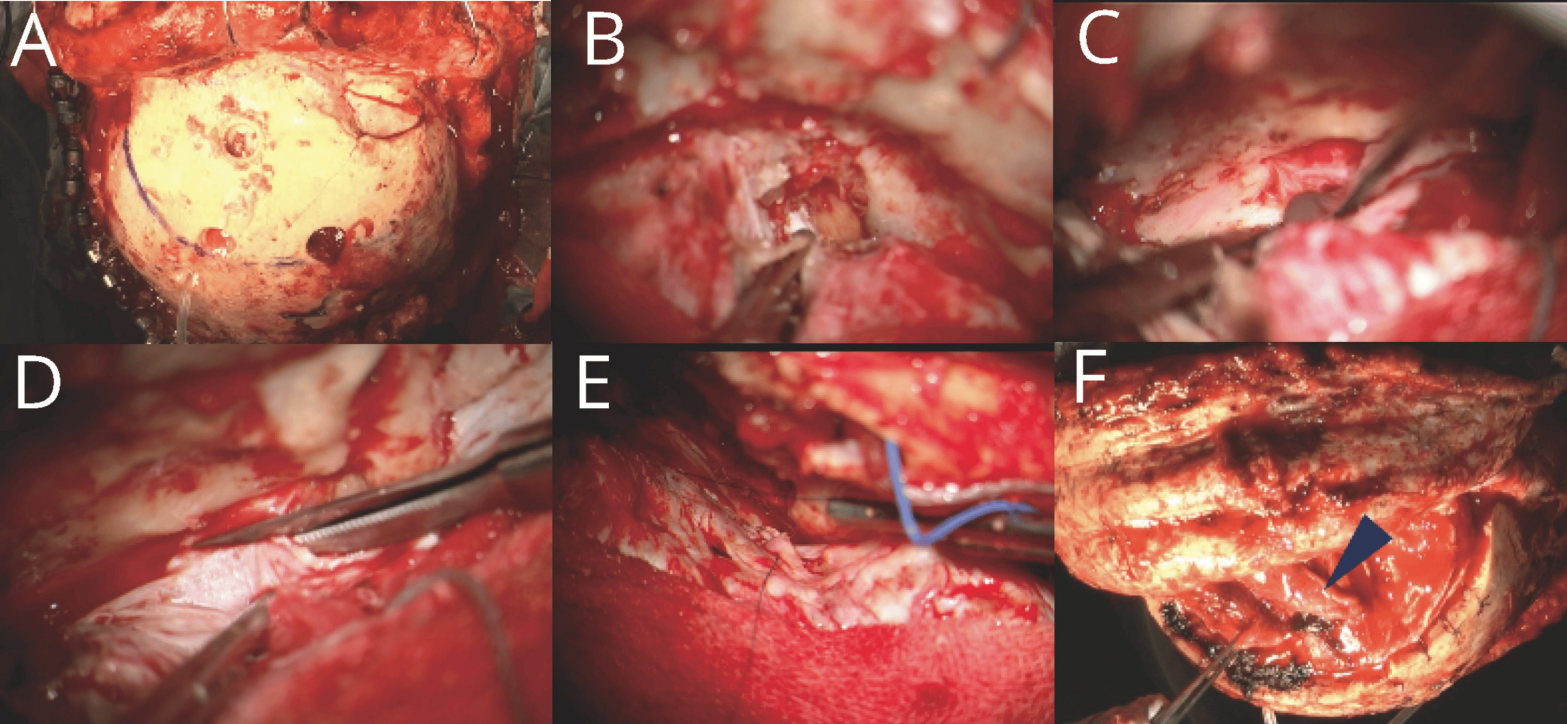
Intraoperative findings of dural repair. A: An intraoperative image was taken during the dural repair. A coronal skin incision was made, and a pedicled periosteal flap was harvested, followed by frontal craniotomy. B: The right frontal lobe was retracted, and the right olfactory nerve was transected intradurally. C: The dura herniated into and adhered to the ethmoid sinus at the bony defect site. D: The dura herniating into the ethmoid sinus was incised. E: The collagen matrix graft was placed in the subdural space, and the dura was sutured using 6-0 PDS suture material. F: A pedicled periosteal flap was placed to cover the bony defect of the anterior skull base. The blue arrow indicates the pedicled periosteal flap.

On day 15, the patient had dural repair surgery. Under general anesthesia, a coronal skin incision was made and a pedicled periosteal flap was harvested. Bifrontal craniotomy (7 × 10 cm) was performed and the frontal lobes were retracted extradurally. The dura at the frontal skull base was dissected and the crista galli were removed. The right olfactory nerve was sacrificed at the dural penetration site (Figures 3A, 3B). The dura and scar tissues at the site of bone separation exhibited strong adhesions and were incarcerated in the ethmoid sinus, making dissection challenging (Figure 3C). After the dura on the ethmoid side was incised (Figure 3D), the contused brain parenchyma was exposed. In addition, as much of the contused brain as possible was removed extradurally. The contused brain turned yellow, resembling pus, suggesting a possible infection. The collagen matrix graft was placed in layers, and the dura was sutured with 6-0 PDS (Ethicon, Inc., Somerville, USA) (Figure 3E). A pedicled periosteal flap was placed over the frontal skull base to block communication with the paranasal sinuses, and the surgery was completed (Figure 3F).

**Figure 4:**
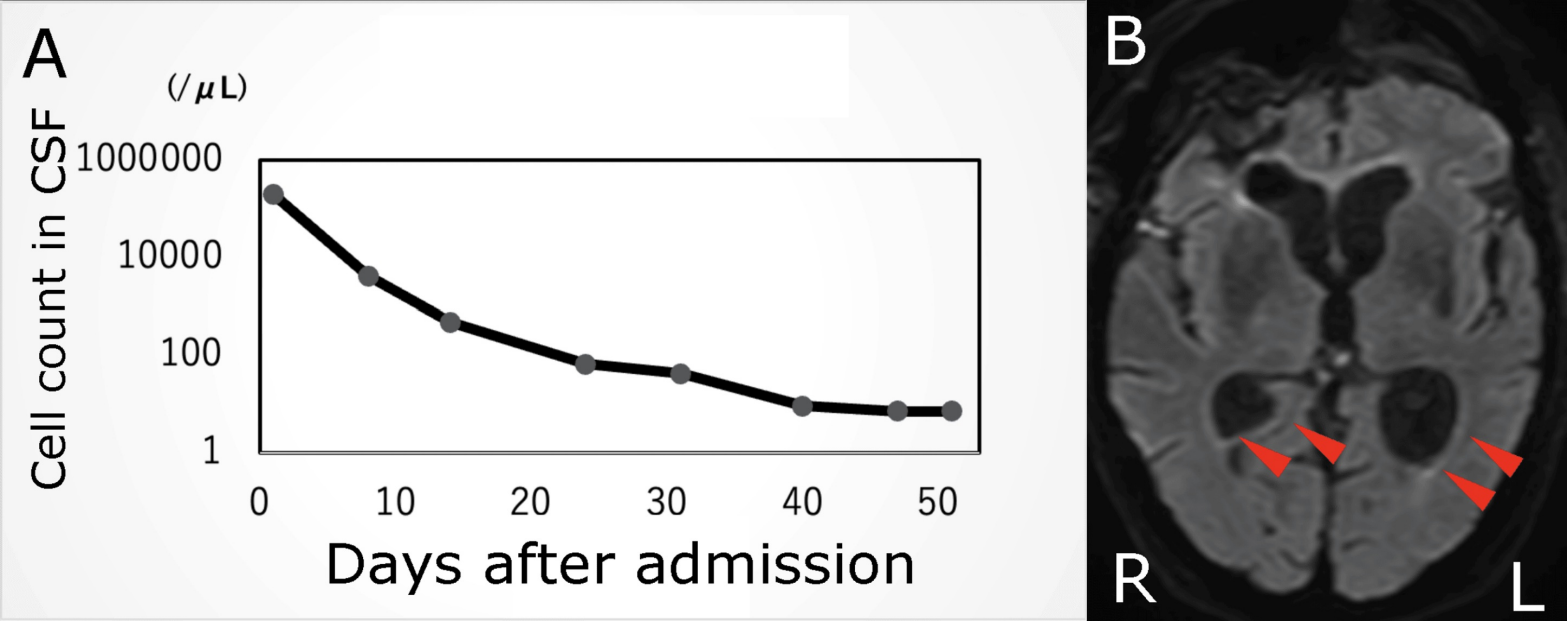
Postoperative CSF and MRI findings. A: Chart showing the course of cerebrospinal fluid cell counts in the patient during hospitalization. B: Axial DWI image taken after antibiotic treatment. The red arrows indicate the disappearance of the high-intensity area within the ventricle. DWI: diffusion-weighted imaging

Postoperatively, the patient continued to receive antibiotics for 31 days after surgery, while the CSF cell count gradually decreased (Figure 4A). Follow-up MRI showed resolution of the hyperintense areas on DWI, and antibiotics were discontinued on day 40 (Figure 4B). On day 71, the patient underwent a lumbar-peritoneal shunt for post-meningitis hydrocephalus and was transferred to a long-term care facility with an mRS score of 5 on day 86.

## Discussion

Herein, we encountered a case of severe delayed-onset bacterial meningitis that developed one year following a frontal skull base fracture, despite the absence of a CSF leak during the post-injury period. CSF leakage occurs when the dura is damaged due to head trauma, particularly in cases involving skull base fractures, at an incidence of 12-30% [9]. CSF leaks can result from communication between the nasal or ear cavities and the intracranial space, leading to meningitis [13]. Approximately 60% of traumatic CSF leaks occur within a few days of head injury, while 95% occur within three months [14]. Traumatic CSF rhinorrhea resolves spontaneously in 50-80% of cases within one to three weeks, while CSF otorrhea resolves within 5-10 days. However, even if the CSF leak resolves, there is a risk of occult CSF leakage and progression to delayed-onset meningitis [15-17].

While delayed-onset posttraumatic meningitis is rare, Kamochi et al. reported one case of meningitis that developed more than 20 years after head trauma, with the patient showing signs of CSF rhinorrhea several years before the onset of meningitis [18]. In our case, despite no evidence of CSF leak during the post-traumatic period, the patient developed delayed-onset meningitis one year after injury. This is a unique feature of the present case.

Large skull base defects resulting from head trauma increase the risk of CSF leakage [19]. In cases of large bony separation, as seen in our patient, hematoma or contused brain tissue may have incarcerated into the fracture site, preventing the manifestation of CSF leakage, despite dural injury. Among skull base fractures, CSF leaks are most commonly associated with frontal skull base fractures [20]. Even in the absence of a CSF leak, potential occult dural injury should be considered in cases of large frontal skull base defects, as in our patient.

In the present case, MRI revealed a new DWI high-intensity area in the right lateral ventricular anterior horn, and endoscopic examination confirmed pus entering the lateral ventricle from contused brain tissue outside the ventricle. This indicated that the dura had been damaged, resulting in communication between the intracranial space and the ethmoid sinus. In the CT scan of the head injury caused by a traffic accident, the hematoma in the frontal lobe contusion did not rupture into the ventricle. The subsequent infection could develop a new communication with the ventricle. Thus, even in the absence of a CSF leak, a thorough investigation of the infection pathway, including invasive testing, is crucial.

Delayed CSF leakage rarely resolves spontaneously because of adhesions to surrounding tissues and is often resistant to conservative treatment [18]. Early dural repair is necessary to prevent further complications [21,4]. In the present case, the entrapped dura and adjacent tissues exhibited strong adhesions, which necessitated surgical intervention to repair the dural defect and block communication between the paranasal sinuses and the intracranial space. Despite a severe infection upon admission, dural repair using a pedicled periosteal flap led to a favorable outcome and was deemed essential in this context. Kamochi et al. also reported the successful treatment of delayed-onset post-traumatic meningitis using dural repair with a pedicled periosteal flap [18]. This technique was chosen over free grafts due to its many advantages, including strong resistance to infection and rapid integration into recipient sites [20]. Surgical intervention is recommended in cases in which delayed-onset meningitis associated with dural injury is suspected.

## Conclusions

Skull base trauma with bone defects in the frontal region can lead to unpredictable, delayed-onset, severe meningitis, even in the absence of CSF leakage. As such, the possibility of occult dural injury should be considered, regardless of the presence of a CSF leak. If meningitis occurs, surgical dural repair should be considered in addition to antibiotic therapy to ensure effective management of the infection.
